# Autosomal dominant optic atrophy: A novel treatment for *OPA1* splice defects using U1 snRNA adaption

**DOI:** 10.1016/j.omtn.2021.10.019

**Published:** 2021-10-21

**Authors:** Christoph Jüschke, Thomas Klopstock, Claudia B. Catarino, Marta Owczarek-Lipska, Bernd Wissinger, John Neidhardt

**Affiliations:** 1 Human Genetics, Faculty of Medicine and Health Sciences, University of Oldenburg, 26129 Oldenburg, Germany; 2 Friedrich-Baur Institute, Department of Neurology, University Hospital, LMU Munich, University of Munich, 80336 Munich, Germany; 3 German Center for Neurodegenerative Diseases (DZNE), 81377 Munich, Germany; 4 Munich Cluster for Systems Neurology (SyNergy), 81377 Munich, Germany; 5 Institute for Ophthalmic Research, Center for Ophthalmology, University of Tübingen, 72076 Tübingen, Germany; 6 Research Center Neurosensory Science, University of Oldenburg, 26129 Oldenburg, Germany; 7 Joint Research Training Group of the Faculty of Medicine and Health Sciences, University of Oldenburg, 26129 Oldenburg, Germany and University Medical Center Groningen, 9700 RB Groningen, the Netherlands

**Keywords:** gene therapy, U1 snRNA, ExSpeU1, splicing, Dominant Optic Atrophy, DOA, Autosomal Dominant Optic Atrophy, ADOA, Optic Atrophy Type 1, OPA1

## Abstract

Autosomal dominant optic atrophy (ADOA) is frequently caused by mutations in the optic atrophy 1 (*OPA1*) gene, with haploinsufficiency being the major genetic pathomechanism. Almost 30% of the *OPA1*-associated cases suffer from splice defects. We identified a novel *OPA1* mutation, c.1065+5G>A, in patients with ADOA. In patient-derived fibroblasts, the mutation led to skipping of *OPA1* exon 10, reducing the OPA1 protein expression by approximately 50%. We developed a molecular treatment to correct the splice defect in *OPA1* using engineered U1 splice factors retargeted to different locations in *OPA1* exon 10 or intron 10. The strongest therapeutic effect was detected when U1 binding was engineered to bind to intron 10 at position +18, a position predicted by bioinformatics to be a promising binding site. We were able to significantly silence the effect of the mutation (skipping of exon 10) and simultaneously increase the expression level of normal transcripts. Retargeting U1 to the canonical splice donor site did not lead to a detectable splice correction. This proof-of-concept study indicates for the first time the feasibility of splice mutation correction as a treatment option for ADOA. Increasing the amount of correctly spliced *OPA1* transcripts may suffice to overcome the haploinsufficiency.

## Graphical abstract

**Figure:**
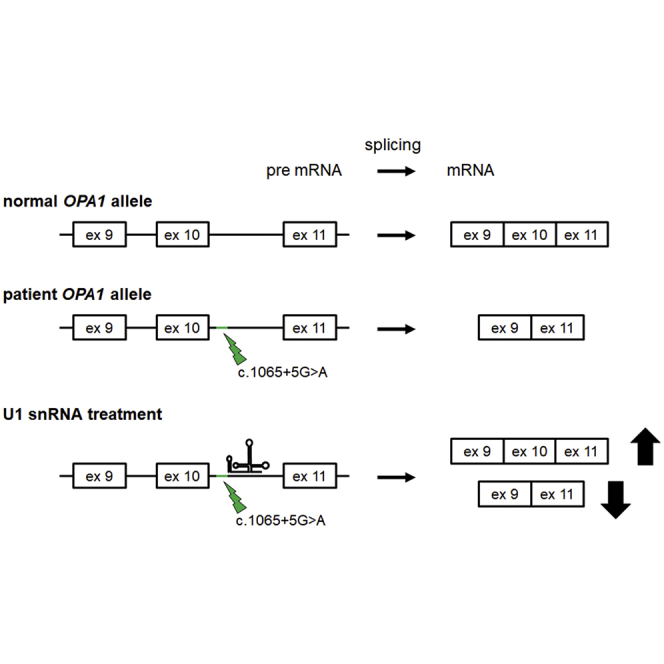

## Teaser

Optic atrophy is one of the most common inherited optic neuropathies, with progressive loss of vision starting in childhood. In patient-derived fibroblasts, the authors identified a mutation in *OPA1* causing aberrant precursor mRNA splicing concomitant with reduced protein expression. Using engineered small nuclear RNAs, they could partially restore normal splicing.

## Introduction

Mutations in the optic atrophy type 1 gene (*OPA1*; OMIM: 605290) cause autosomal dominant optic atrophy (ADOA; OMIM: 165500), which is characterized by slowly progressive bilateral loss of visual acuity, centrocecal visual field defects, and color vision disturbances. The disease typically starts during early childhood, and most patients present with optic disc pallor indicating bilateral atrophy of the optic nerve due to degeneration of retinal ganglion cells. Nevertheless, high intra- and interfamilial variability in the degree of visual impairment in ADOA occurs, ranging from normal vision to legal blindness. With a disease prevalence of about 1:10,000 to 1:30,000, ADOA is one of the most common inherited optic neuropathies.^1^

The *OPA1* gene is a nuclear gene that encodes a protein of the inner mitochondrial membrane with similarity to dynamin-related GTPases.^2^^,^^3^ Co- or post-transcriptional processing, i.e., alternative splicing, and post-translational processing, i.e., proteolytic cleavage, generate a series of long (L-OPA1) and short isoforms (S-OPA1), the exact balance of which is essential for proper OPA1 functioning.^4^, ^5^, ^6^, ^7^, ^8^, ^9^ On the cellular level, OPA1 is critically involved in several processes, both affecting and depending on the integrity of the mitochondrial inner membrane, such as inner mitochondrial membrane fusion, crista maintenance, cell survival, mitochondrial energetics, and genome stability.^10^, ^11^, ^12^

Mutations leading to erroneous precursor-mRNA splicing represent a significant proportion of pathogenic human genome alterations. Approximately 10%–20% of human disease-causing mutations affect canonical splice sites and lead to splice defects (Human Gene Mutation Database [HGMD], http://www.hgmd.cf.ac.uk/ac/index.php),^13^ often associated with severe clinical phenotypes. Furthermore, up to 50% of mutations are estimated to affect splicing by disrupting the splicing code, i.e., affecting not only consensus splice sites, but also splice-regulatory elements, altering secondary structure or creating cryptic splice sites.^14^^,^^15^

Correct splicing of nuclear pre-mRNA depends on a complex interplay of different splicing factors.^16^ The first steps require the identification of the exonic sequences within the large pre-mRNA and the exact recognition of exon-intron boundaries. Many splicing factors need to recognize *cis*-acting elements within the pre-mRNAs. Spliceosome formation is initiated by the small ribonucleoprotein particle U1 (U1 snRNP), composed of multiple proteins and a 164 nt long non-coding RNA, which recognizes the splice donor site (5′ splice site). The consensus sequence of the splice donor site consists of a nine-nucleotide sequence, which is recognized by the 5′ end of U1 through complementary base pairing covering the first six nucleotides of the 5′ end of the intron (+1 to +6) and the last three nucleotides of the 3′ end of the exon (−3 to −1). Binding of U1 initiates the splicing process and the recruitment of further splice factors.^16^ However, if mutations alter the splice donor site, efficient binding of U1 may be impaired, usually leading to aberrant splicing. While the different positions within the splice donor site are neither equally conserved nor functionally equivalent, a minimal number of base pair matches with U1 seem to be required to ensure proper splicing.^17^^,^^18^

In recent years, we and others have developed strategies to restore correct splicing of disease-causing splice donor site mutations by modifying the 5′ tail of U1, with the aim to match exactly to the mutated splice donor site by base-pairing interaction. This approach proved to be successful in many cases.^19^, ^20^, ^21^, ^22^, ^23^, ^24^

Studies using minigenes have established that correct splicing can be reconstituted by targeting engineered U1s not only directly toward the mutated consensus site, but also to the proximity of the exon-intron junction.^25^ Engineered U1s with 5′ tails binding to intronic sequences downstream of the affected exon also showed efficacy to correct mutation-induced splice defects in several model systems.^25^, ^26^, ^27^, ^28^

In this study, we report the identification of a family with ADOA from Germany harboring a novel splice donor site mutation in the *OPA1* gene. Analyses in patient-derived cells showed that exon skipping in mutant transcripts reduced OPA1 protein levels. In support of our results, a mouse model of optic atrophy harboring the homologous sequence alteration in the mouse *Opa1* gene showed similar mutation-induced effects on transcript and protein levels.^29^ We developed a genetic therapy to correct the mutation-induced splice defect applying engineered U1 and demonstrating that the U1 splicing factor, fully adapted to a region downstream of the splice donor site in *OPA1* intron 10, is able to significantly ameliorate correct splicing of *OPA1* in a dose-dependent manner in patient-derived fibroblasts.

## Results

### Clinical reports of an ADOA family

#### Index patient

**Figure 1:**
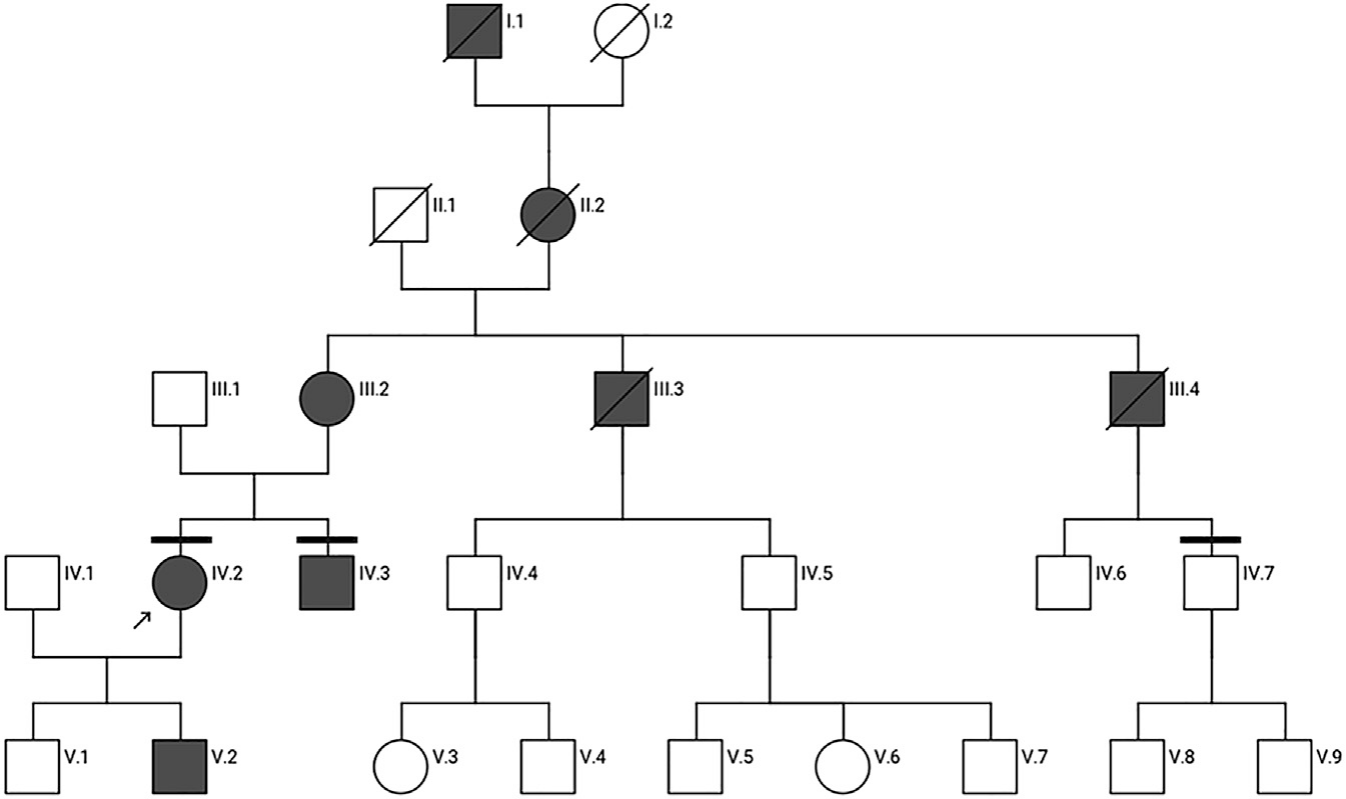
Pedigree of the family with several affected members in five consecutive generations with autosomal dominant optic atrophy The index patient IV.2 (indicated by an arrow) and seven other affected family members in five generations suffered from slowly progressive bilateral visual impairment and optic atrophy. For individuals I.1 and II.2, visual impairments were reported only in the family history. Family members III.2, IV.2, and IV.3 were seen in clinic and diagnosed with *OPA1*-associated ADOA. Horizontal bars mark subjects (IV.2, IV.3, and IV.7) who were genetically analyzed and donated skin biopsies, from which patient cell lines were derived. Circles represent females, squares represent males. Filled symbols represent individuals affected with ADOA; diagonal lines indicate deceased individuals.

The index patient (IV.2) is the eldest of two siblings from a non-consanguineous family from Germany (Figure 1). Disease onset was during childhood in the primary school years and a slowly progressive bilateral visual loss was documented. There is no other relevant medical history. At the age of presentation in clinic, she was 34 years of age and had a moderate bilateral visual loss (best corrected visual acuity [BCVA] OD/OS 0.25), temporal predominant optic pallor, and exophoria in the cover test. General and neurological examination were otherwise unremarkable.

#### Patient IV.3

The affected brother (IV.3) of the index patient had clinical onset in preschool years (kindergarten) and had surgery for strabismus at the age of 5 (Figure 1). At the age of 32 years, he presented with slowly progressive bilateral visual loss, with a visual acuity of OD 0.20 and OS 0.16, temporal predominant optic pallor, and exophoria. Furthermore, dyschromatopsia and a centrocecal visual field defect were observed, as well as a peripapillary reduced thickness of the retinal nerve fiber detected by optical coherence tomography (OCT) imaging. General and neurological examination were otherwise unremarkable.

#### Patient III.2

The affected mother (III.2) of the index patient had clinical onset at the age of 15 years, with bilateral visual impairment, slowly progressive over the years (Figure 1). Optic atrophy was documented. In addition, neurological examination showed spastic paraparesis, pallhypesthesia, and spastic-ataxic gait.

#### Subject IV.7

The first cousin (IV.7) of the index patient was asymptomatic (Figure 1). His general, neurological, and ophthalmological examination was unremarkable.

Further details of the clinical characterization for the remaining affected family members, who were not seen in clinic, are summarized in Table S1 and included in the supplementary information.

### Identification of the sequence variant c.1065+5G>A in *OPA1*

Complete sequencing of the mitochondrial DNA showed no pathogenic variants in the index patient. Her mother (III.2) underwent genetic testing using an optic atrophy gene panel, which revealed a heterozygous sequence variant in the *OPA1* gene, c.1065+5G>A in intron 10 (NM_015560.2), initially classified as a variant of uncertain significance (VUS; VUS class 3 of the American College of Medical Genetics and Genomics classification).^30^ Mutation analysis by Sanger sequencing confirmed the presence of the heterozygous *OPA1* mutation, while it was absent in the healthy first cousin (IV.7, Figure 1) of the index patient. These results suggested co-segregation of the mutation with the disease. Notably, the homologous mutation c.1065+5G>A in the murine *Opa1* gene is present in a mouse model for ADOA,^29^ further supporting the pathogenicity of the identified *OPA1* variant in our family.

### Genetic studies in patient-derived cell lines

**Figure 2:**
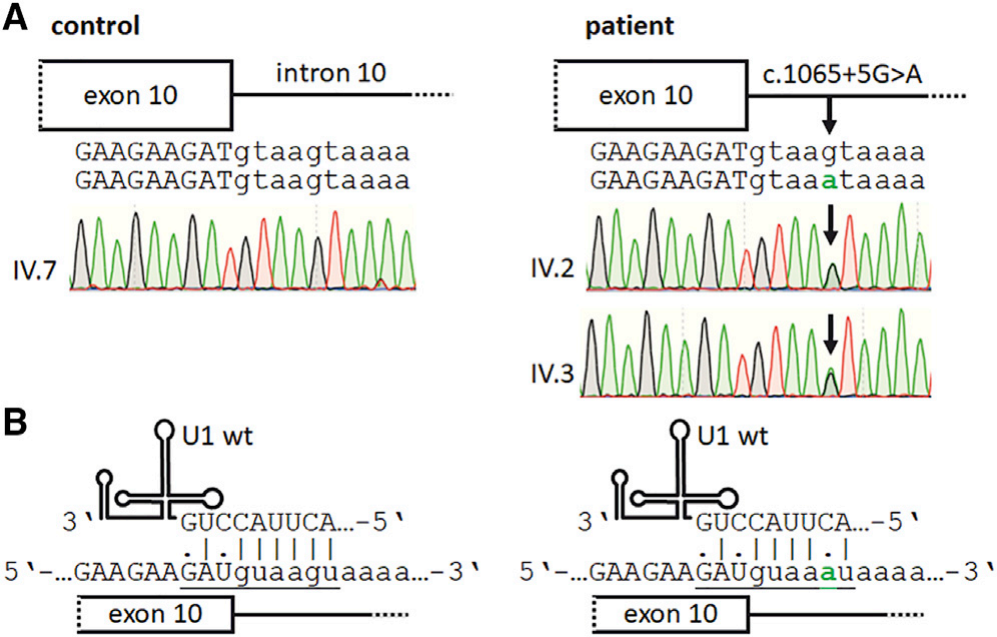
Identification of a novel human *OPA1* mutation, c.1065+5G>A, affecting the splice donor site of exon 10 (A) Sanger sequencing profiles of family members IV.7 (unaffected), IV.2 (affected), and IV.3 (affected). The electropherograms show the splice donor site of *OPA1* exon 10 as well as flanking genomic sequences. The arrows indicate the heterozygous *OPA1* mutation c.1065+5G>A. Exonic nucleotides are shown in capital letters, intronic nucleotides in lowercase letters. The mutated nucleotide is highlighted in green. (B) The *OPA1* mutation c.1065+5G>A is likely to interfere with the recognition of the splice donor site by the wild-type U1 small nuclear RNA (U1 wt). The G>A sequence alteration reduces the complementary base pairing interaction between the U1 and the exon 10 splice donor site from 7 to 6 bp. The mutated nucleotide is highlighted in green. The nucleotides of the splice donor site are underlined.

To investigate the molecular consequences of the *OPA1* mutation, we generated patient-derived cell lines from skin biopsies of the index patient (IV.2), her affected brother (IV.3), and one healthy first cousin (IV.7). Sanger sequencing of genomic DNA isolated from skin fibroblasts of the two affected siblings confirmed the heterozygous mutation *OPA1*: c.1065+5G>A, whereas the patient-derived cells of the healthy cousin (IV.7) did not show the mutation (Figure 2A). The mutation is predicted to reduce the affinity between the 5′ end of the splicing factor U1 and the splice donor site of *OPA1* exon 10 (Figure 2B). The complementarity between the U1 and the splice donor site is reduced by one base pairing, a sequence alteration that has the potential to induce splice defects in the *OPA1* transcript. Of note, the base pair at the +5 position is among the most conserved nucleotides of the splice donor site. Binding of the U1 is an essential step for initiation of spliceosome formation. Furthermore, the mutation abolishes a predicted binding site of the splice enhancer SRSF6, which may be required here for proper splicing in addition to U1 (Figure S1).

### *OPA1* transcript analysis reveals exon 10 skipping in the mutated allele

**Figure 3:**
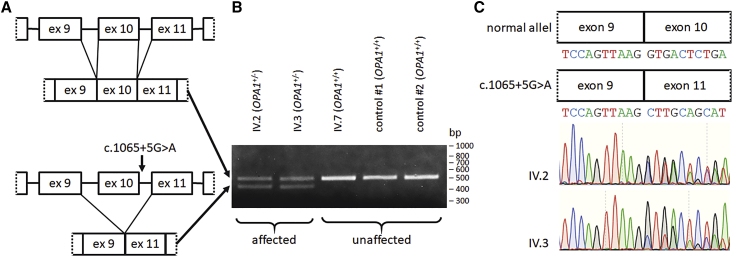
*OPA1* mutation c.1065+5G>A leads to exon 10 skipping (A) Schematic drawing of the reference and the mutated *OPA1* allele. The two alleles generate splicing products that either include or skip exon 10. (B) Skipping of *OPA1* exon 10 during splicing leads to an 81 nt deletion in the *OPA1* transcript. Total RNA from cultivated skin fibroblasts was analyzed by RT-PCR with primers amplifying *OPA1* exons 7 to 13. The cDNA from controls 1 and 2 was generated from primary fibroblasts donated by unrelated healthy individuals. All cell lines were cultured under highly comparable conditions. (C) Sequencing confirms exon 10 skipping in the *OPA1* transcript of patient cell lines. Direct sequencing of RT-PCR products from family members IV.2 and IV.3 reveals an overlapping sequence starting after exon 9 due to exon 10 skipping in the mutant *OPA1* transcript. Overlapping sequences were subtracted from each other to confirm *OPA1* exon 10 skipping in the mutated allele. Total RNA was isolated from patient-derived skin fibroblasts.

We asked whether the c.1065+5G>A mutation may affect the *OPA1* transcript splicing process. We selected primer binding sites to distinguish between the correctly spliced *OPA1* transcript, resulting in an RT-PCR product of 500 bp, and the transcript skipping exon 10, leading to a shortened product of 419 bp. The healthy control IV.7 exclusively showed the RT-PCR product corresponding to the full-length *OPA1* transcript. In contrast, almost equal amounts of the full-length (including exon 10) and the shortened (skipping exon 10) RT-PCR product were detected in the patient-derived cell lines of IV.2 and IV.3 (Figures 3A and 3B). This indicated that the mutation c.1065+5G>A is highly penetrant to induce skipping of exon 10 in mutated *OPA1* transcripts. It further suggested that both alleles in the patients were transcribed at approximately equal levels. RT-PCR products were confirmed by sequencing (Figure 3C). Comparably, the homologous mutation in the mouse *Opa1* gene caused skipping of exon 10 and showed almost equal expression levels between the mutated and the wild-type transcripts.^29^

### Reduced OPA1 protein expression in patient-derived cells

**Figure 4:**
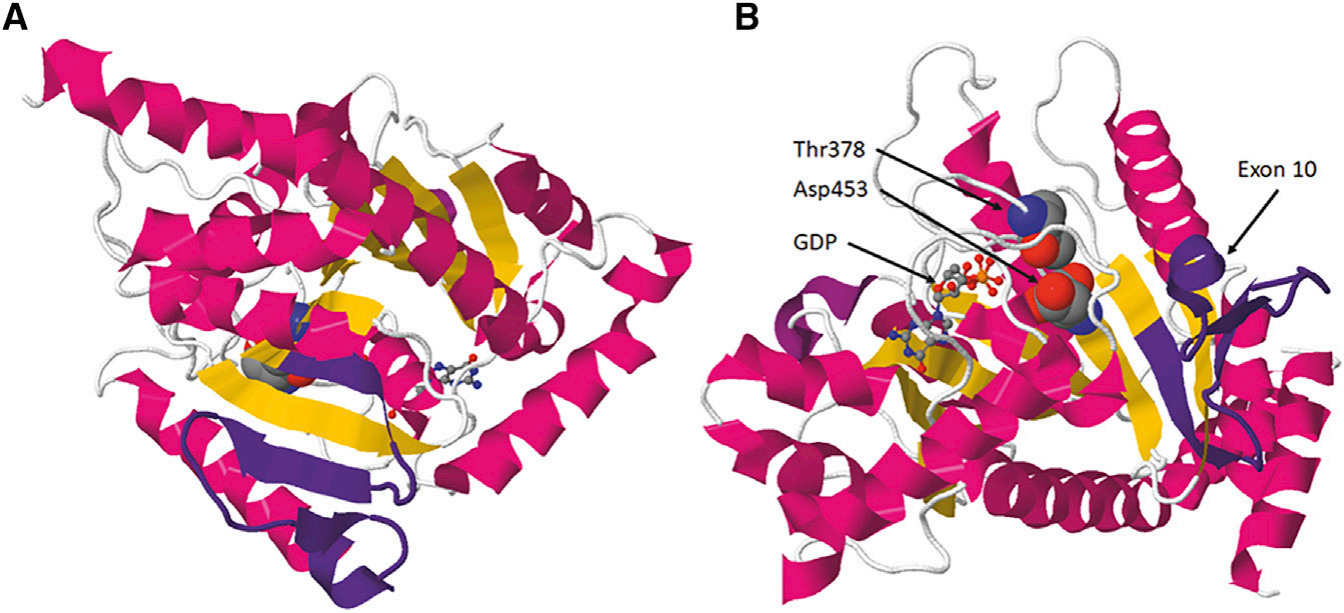
Structural model of the GTPase domain of OPA1 highlighting exon 10 Three-dimensional representation of the minimal GTPase domain of OPA1 (PDB: 6JTG^31^). (A) Frontal view of the amino acids encoded by exon 10 (shown in indigo) within the GTPase domain of OPA1. (B) View of the structural model showing the GDP-bound form of the GTPase domain of OPA1. Amino acids involved in GTPase function are highlighted. Asp453Ala or Thr378Ala substitution drastically reduces the GTPase activity of OPA1.^31^ The model suggests that exon 10-encoded amino acids might fulfill a structural role in positioning Thr378 and Asp453 toward the GDP β-phosphate. The α helices are shown in red, β strands are shown in yellow, GDP is represented as a ball-and-stick model, and the amino acids Thr378 and Asp453 are represented as space-filling models.

Exon 10 skipping causes the loss of 81 nucleotides in *OPA1* transcripts, leading to an in-frame deletion of 27 amino acids (VTLSEGPHHVALFKDSSREFDLTKEED, NP_570850.2: p.(Val384_Asp410del)) in the OPA1 GTPase domain. The affected amino acids are part of highly conserved anti-parallel β sheets and an α helix (Figures 4 and S2). The structural models of the OPA1 GTPase domain suggested that a deletion of exon 10-encoding amino acids mainly affects the β sheets running through the center of the OPA1 GTPase domain. It seems possible that this might result in either unstable or misfolded OPA1 (Figures 4 and S3). Of note, p.Asp453Ala or p.Thr378Ala substitutions in the GTPase region were sufficient to drastically reduce the catalytic GTPase activity of OPA1,^31^ probably by interfering with the coordination of a Mg^2+^ ion, further supporting the notion that improper protein folding might impair the function of the OPA1 GTPase domain (Figure S3).

**Figure 5:**
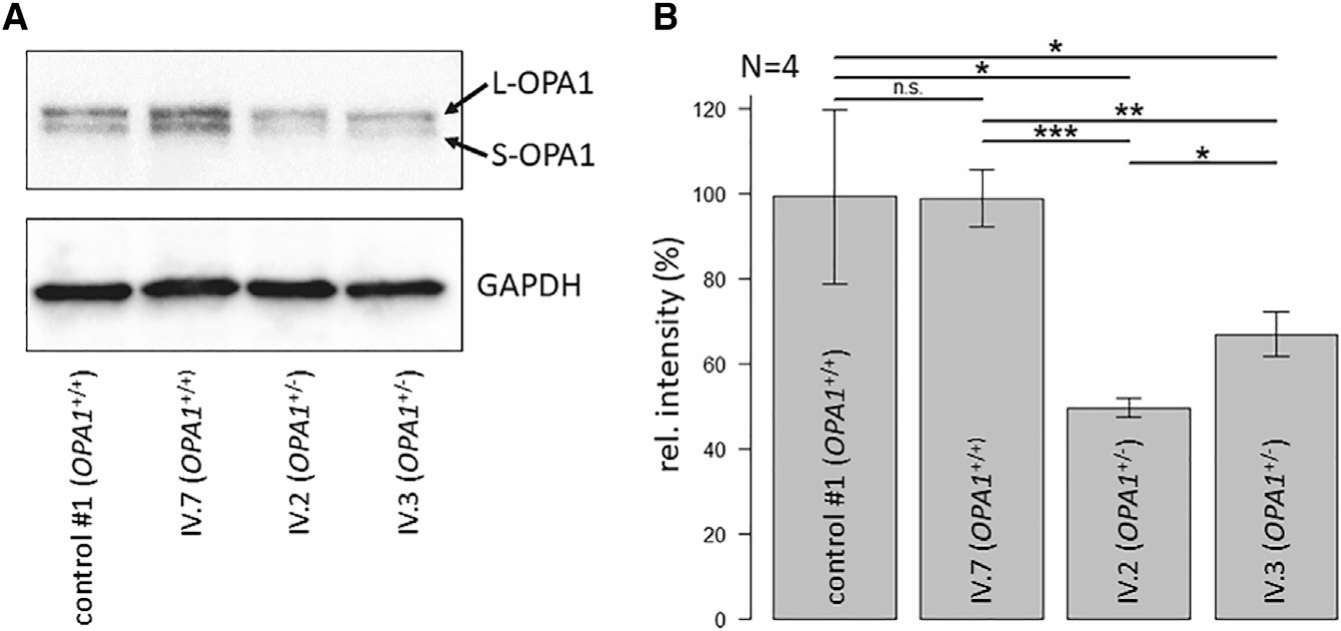
Western blot analysis and quantification of OPA1 protein expression (A) Western blot for the determination of OPA1 levels in primary fibroblasts from patients and controls. OPA1 appears as two bands, L-OPA1 and S-OPA1. GAPDH was used as a loading control. (B) Relative quantification of OPA1 protein levels by densitometric analyses. The level of OPA1 was normalized against GAPDH. Control 1 is an unaffected individual from an unrelated family. Error bars indicate the SD from four biological replicates (n = 4). Statistical significances were calculated by pairwise t tests with non-pooled SD. ∗∗∗p < 0.001; ∗∗p < 0.01; ∗p < 0.05; n.s., not significant, p > 0.05.

We asked whether the reduction in the correctly spliced *OPA1* transcript affects steady-state levels of the OPA1 protein. Previously, we observed that the correlation between transcript and protein levels can vary significantly for different genes.^32^ For *OPA1*, we determined that native OPA1 protein levels were reduced by approximately 50% in patient-derived cells compared with controls (IV.7 versus IV.2, p = 0.00027; IV.7 versus IV.3, p = 0.00039), resembling the reduction in correctly spliced transcript levels (Figures 3 and 5). The reduction in full-length *OPA1* transcript levels was not compensated for, neither by increased translation rates nor by other post-transcriptional regulatory mechanisms. We did not detect shortened OPA1 protein forms in the patient samples, indicating that the misspliced transcripts were either untranslated or resulting in unstable and/or quickly degraded OPA1 proteins. Our results closely resemble the western blot analyses of Opa1 protein expression in the homologous mouse model, as well as in mouse embryonic fibroblasts (MEFs) derived from this mouse model.^29^ Interestingly, the two affected siblings from the family showed a significant difference in OPA1 protein levels (IV.2 versus IV.3, p = 0.0039).

Taken together, our data showed that the mutation c.1065+5G>A leads to exon 10 skipping and suggested a close correlation between transcript and protein expression in *OPA1*. While both alleles were transcribed, a protein product originating from the mutated transcript could not be identified. Compensatory increase in the translation of the correct allele was not suggested by our data.

### Therapeutic correction of *OPA1* exon 10 skipping by applying engineered U1 small nuclear RNA (snRNA)

The majority of *OPA1* mutations associated with ADOA are caused by the molecular mechanism of haploinsufficiency.^33^ Consequently, increasing *OPA1* expression represents a promising therapeutic approach to treat *OPA1*-associated diseases. The mutation described herein adds to this category of haploinsufficiency, as it most likely is a loss-of-function mutation and leads to approximately 50% reduction in protein expression. Importantly, proper OPA1 function depends on the delicate balance of different L- and S-OPA1 isoforms. We aimed to convert misspliced *OPA1* transcripts into correctly spliced *OPA1* transcripts, i.e., increase the fraction of functional *OPA1* transcripts without interfering with the processing of isoforms. To reach this goal, we applied engineered U1s to compensate for the reduced binding affinity between the normal U1 and the mutated *OPA1* transcripts.

**Figure 6:**
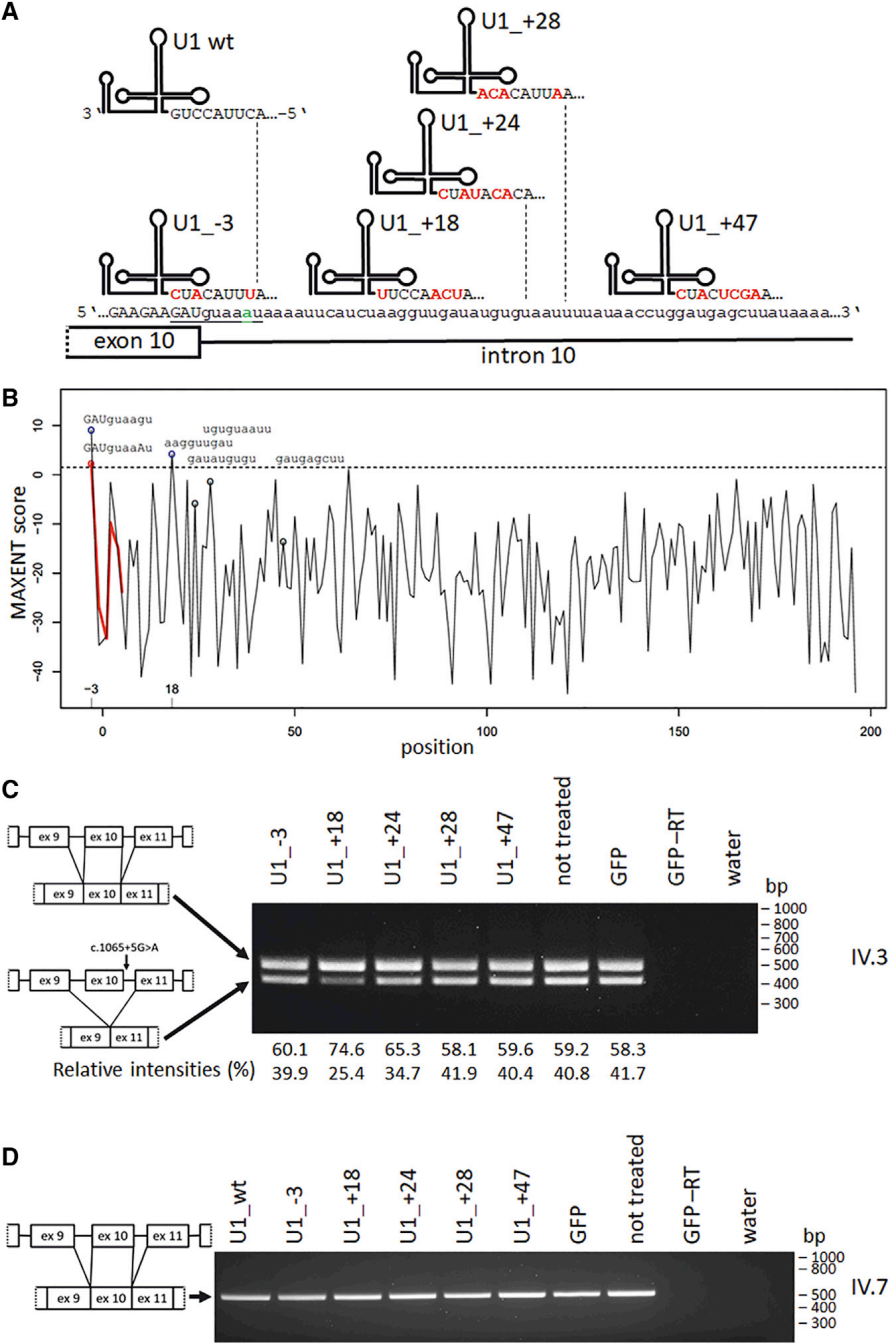
Design and screening of engineered U1s (A) Schematic diagram of engineered U1s showing their potential binding sites at the genomic region of the splice donor site of *OPA1* exon 10/intron 10. Nucleotides that were changed within the 5′ part of the U1 snRNA are highlighted in red. Five different engineered U1s were designed to bind either to the mutated splice donor site or to distinct intronic sequences downstream of exon 10. Nucleotides of the splice donor site are underlined; the *OPA1*: c.1065+5G>A mutation is highlighted in green. (B) Maximum entropy model (MAXENT) scores of potential splice donor sites in *OPA1* intron 10 in comparison with the mutated *OPA1*: c.1065+5G>A allele. The *OPA1*: c.1065+5G>A mutation causes the +18 position (aaggttgat; 18 bp downstream of the exon/intron 10 boundary in *OPA1*) to become the position with the highest MAXENT score. The mutation decreased the MAXENT score of the reference splice donor site (−3 position) by 6.82 units (from 9.11 to 2.29). The red line highlights the differences in the results of the MAXENT algorithm for the *OPA1*: c.1065+5G>A mutation. Positions are numbered relative to the exon/intron boundary. Sequences exhibiting MAXENT scores above 1.5 are indicated by blue circles. Their potential splice donor sites are shown. Sequences with scores below 1.5 are indicated by black circles (http://hollywood.mit.edu/burgelab/maxent/Xmaxentscan_scoreseq.html).^35^ Exonic nucleotides are shown in capital letters, intronic nucleotides in lowercase letters. (C) RT-PCR analysis of *OPA1* splice events after treatment with engineered U1 snRNAs. Primary dermal fibroblasts from patient IV.3 were treated with lentiviral vectors expressing five engineered U1 snRNAs (U1_−3, U1_+18, U1_+24, U1_+28, U1_+47; compare with [A]), and GFP only. Total RNA from cultivated fibroblasts was analyzed by RT-PCR with primers amplifying *OPA1* exons 7 to 13. The engineered U1 snRNA U1_+18 led to a detectable reduction in the misspliced *OPA1* transcript. For both patient-derived cell lines (IV.2 and IV.3), these analyses were independently repeated. (D) Treatment of control fibroblasts with different adapted U1 snRNAs. Primary dermal fibroblasts from the control IV.7 were treated with lentiviral vectors expressing wild-type U1 snRNA (U1_wt), five engineered U1 snRNAs (U1_−3, U1_+18, U1_+24, U1_+28, U1_+47), and GFP only. Total RNA from cultivated fibroblasts was analyzed by RT-PCR with primers amplifying *OPA1* exons 7 to 13. GFP, construct expressing only GFP; GFP −RT, cDNA reaction without reverse transcriptase.

We and others have previously shown that splice defects induced by mutations in splice donor sites can be partially corrected by overexpression of engineered U1.^19^, ^20^, ^21^, ^22^, ^23^, ^24^^,^^34^ We tested this therapeutic approach on the mutation described herein and designed an engineered U1 variant with full complementarity to the mutated *OPA1* splice donor site of exon 10 (Figure 6A). Furthermore, we searched for potential U1 binding sites within the first 60 nt of intron 10. We focused on regions that are low in AU content and exhibit high sequence complexity. We employed the maximum entropy model (MAXENT) algorithm to select potential binding sites of engineered U1.^35^ For the *OPA1*: c.1065+5G>A mutated transcript, the MAXENT score predicted the highest probability for a splice donor site at position +18 of intron 10 (Figure 6B). In total, we generated a series of five different engineered U1 constructs and compared their efficacy in reducing the splice defect in *OPA1* transcripts (Figure 6C).

To assess the therapeutic potential of the five engineered U1s, we transduced patient-derived and control fibroblasts with lentiviral shuttles. Total RNA was isolated and splicing of *OPA1* transcripts analyzed by RT-PCR. In agreement with the MAXENT bioinformatic prediction, we observed a detectable reduction of exon 10 skipping predominantly with the treatment applying U1 constructs directed toward the +18 position in intron 10. For both patient-derived cell lines (IV.2 and IV.3) these results were reproducible, suggesting a successful treatment of the *OPA1* splice defect with engineered U1_+18 (Figure 6C). Western blot analyses indicated a trend toward increased OPA1 protein expression upon treatment with engineered U1_+18 (Figure S4). In contrast, U1 fully adapted to the mutated splice donor site (U1_−3) did not lead to a detectable reduction of the misspliced *OPA1* transcripts. Control fibroblasts showed unaltered *OPA1* expression under all conditions, indicating that the treatment does not interfere with the splicing of *OPA1* reference alleles (Figure 6D).

Taken together, our data demonstrate that the U1s can successfully be engineered to mediate gene therapeutic effects in *OPA1*.

### Dose dependency of the treatment effect

**Figure 7:**
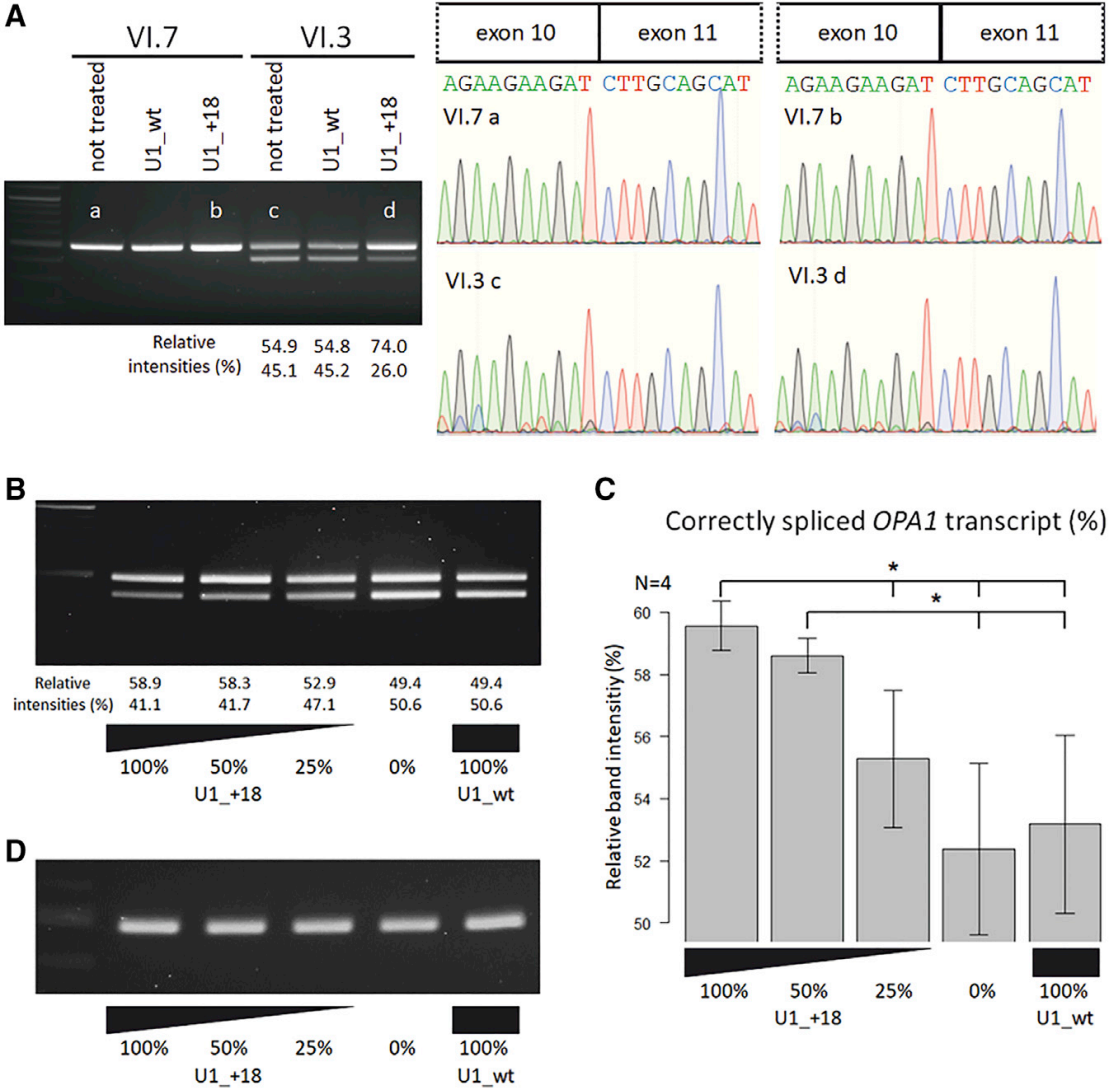
Dose-response effects of the U1 snRNA treatment (A) RT-PCR and sequencing analysis of *OPA1* splice correction in control (VI.7) and patient-derived cell lines (IV.3) after treatment with engineered U1 snRNAs. Total RNA from cultivated fibroblasts was analyzed by RT-PCR with primers amplifying *OPA1* exons 7 to 13. Gel bands (indicated with a–d) were extracted and sequenced. U1 binding into intron 10 does not affect the correct splice site selection. (B) Patient-derived fibroblasts were treated with a serial dilution of lentiviral shuttles expressing the engineered U1 isoform U1_+18 (100%, 50%, 25%, 0% virus suspension) or U1_wt (100%) as a control. Total RNA from cultured skin fibroblasts was analyzed by RT-PCR with primers amplifying *OPA1* exons 7 to 13. Construct U1_+18 led to a dose-dependent reduction of misspliced *OPA1* transcripts. (C) Relative quantification of correctly spliced *OPA1* mRNA levels (corresponding to the upper bands in [A]) by densitometric analysis. Error bars indicate the SD from four biological replicates (n = 4). ∗p < 0.05. (D) Control fibroblasts were treated with a serial dilution of lentiviral shuttles expressing the engineered U1 isoform U1_+18 (100%, 50%, 25%, 0%) or U1_wt (100%) as a control. Total RNA was analyzed by RT-PCR with primers amplifying *OPA1* exons 7 to 13. No differences in the splicing of normal *OPA1* transcripts due to the treatment with engineered U1_+18 were observed.

To exclude the potential activation of cryptic splice donor sites caused by intronic binding of U1_+18 and to verify the correct splicing of the restored *OPA1* transcript, we transduced patient-derived and control fibroblasts with lentiviral shuttles and analyzed splicing of *OPA1* transcripts by RT-PCR (Figure 7A). Gel bands corresponding to the corrected transcripts (Figures 7A, a–d) were extracted and directly sequenced. Sequencing confirmed correct splicing of *OPA1* transcript.

To validate the U1_+18 treatment and to test whether the treatment exhibits a dose-dependent response, we treated patient-derived fibroblasts with a serial dilution (1:2) of lentiviral vectors expressing the U1_+18 construct (Figure 7). Compared with the controls (0% U1_+18- and 100% U1_wt-treated samples), the U1_+18-treated samples showed significant therapeutic efficacy. We observed a dose-dependent treatment effect. The highest dose (100% U1_+18) significantly treated the missplicing of *OPA1*. The treatment inhibited exon skipping and simultaneously increased the expression of full-length *OPA1* transcript compared with the lowest dose (25% U1_+18, p = 0.024), the mock treatment (0%, p = 0.011), and the wild-type treatment (100% U1_wt, p = 0.017). The second highest dose (50% U1_+18) still led to a significant treatment effect compared with the mock treatment (0%, p = 0.019) and the treatment with wild-type U1 (100% U1_wt, p = 0.030). The splice correction efficacy seemed to reach saturation for the two highest concentrations of the lentiviral vector, as they did not show significant differences (100% versus 50% U1_+18, p = 0.104) (Figure 7C).

The treatment with wild-type U1 did not exhibit any effect on correctly spliced transcripts compared with mock treatment (0% versus 100% U1_wt, p = 0.702, Figure 7C). Control fibroblasts showed unchanged *OPA1* expression, independent of the concentration of lentiviral shuttles (Figure 7D).

## Discussion

In this study, we characterized a family with several members suffering from ADOA caused by a novel splice-site mutation in *OPA1* and developed a therapeutic genetic approach to treat the mutation-induced splice defect.

The novel *OPA1*: c.1065+5G>A mutation affects the consensus splice donor site of exon 10 and causes exon 10 skipping during splicing. This single base pair exchange most likely prevents the recognition of the mutated splice donor site by U1, an essential splice factor required to initiate the splicing process. As a consequence, exon 9 of *OPA1* is directly fused to exon 11, resulting in a transcript with an 81 nt in-frame deletion predicted to be translated into an OPA1 protein lacking 27 amino acids. While the shortened transcript is produced at about equal levels compared with the full-length transcript, indicating that both alleles are equally transcribed, we have not been able to detect a shortened OPA1 protein. Instead, we found OPA1 protein levels reduced by approximately 50%. Since exon 10 encodes an essential part of the highly conserved GTPase domain of OPA1, i.e., parts of an α helix and the central anti-parallel β sheet, our data suggested that the shortened protein is most likely non-functional and/or unstable and was degraded rapidly. This supports previous observations that *OPA1* mutations cluster within the GTPase domain and that haploinsufficiency represents the major pathomechanism leading to ADOA.^33^ Our results are in line with previous reports from a mouse model of optic atrophy that carries the homologous c.1065+5G>A *Opa1* mutation, in which both wild-type and mutated transcripts were expressed, and Opa1 protein levels were reduced by approximately 50%.^29^ It can be expected that an increase in reference *OPA1* transcript levels will translate into increased OPA1 protein concentrations.

Clinically, the affected family members presented with typical ADOA symptomatology, including slowly progressive bilateral visual loss, optic atrophy, color vision problems, and cecocentral visual field defects. Some affected family members had other neurological comorbidities concomitantly, such as spastic paraparesis and parkinsonism, as described in other *OPA1*-associated ADOA families. Intrafamilial variability in the age of onset, severity of visual impairment, and presence or absence of other neurological comorbidities was documented, in agreement with previous reports in the literature.^1^ Genetic modifiers and environmental factors have been proposed as an explanation for the complex genotype-phenotype correlations in ADOA caused by *OPA1* mutations.^36^ Our western blot results support these clinical reports on a molecular level, as we were able to quantify significant differences in OPA1 protein expression between the affected siblings (IV.2 versus IV.3, p = 0.0039, Figure 5). However, we were not able to correlate disease severity of our patients with reduced OPA1 protein expression, indicating that OPA1 levels are not the only determinant for ADOA severity. Therefore, additional stratifications will be necessary to correlate OPA1 protein level to ADOA clinical progression or severity. To date, there are no studies published analyzing a potential correlation of OPA1 protein to sex. As our patients were both male and female, we can only speculate that gender may be an important stratification factor, which might warrant investigation in a larger study. Since retinal ganglion cells, which are primarily affected by ADOA, are not accessible for molecular investigations, we cannot exclude that the patient-derived fibroblasts, which we used as a disease model, may not fully resemble OPA1 processing of the retina and optic nerve.

More than 90% of human protein-coding genes generate multiple mRNA isoforms, mostly by alternative splicing. This pre-mRNA processing is frequently affected by disease-causing mutations. It has been estimated that up to 15% of mutations lie within consensus splice sites.^13^ In addition, about 20% of the missense mutations affect predicted splice regulatory elements.^37^

We developed a novel treatment approach for *OPA1* splice defects in primary patient-derived fibroblasts by applying engineered U1 splice factors. We designed U1s with a modified 5′ part binding to different locations in *OPA1* exon 10 or intron 10. With the most efficient engineered U1 constructs, we were able to reduce the mutated transcripts and simultaneously increase the expression of the reference *OPA1* transcripts including exon 10.

RNA splicing and protein processing contribute to different OPA1 isoforms. Normal mitochondrial function depends on the correct ratio between the different OPA1 isoforms.^38^ For gene therapy approaches based on gene augmentation, this poses the challenge of how to maintain or restore the proper ratios of the different isoforms.

We have chosen to engineer U1s to correct the splicing defect in *OPA1*. Compared with gene augmentation strategies, therapeutic U1s have several important advantages: U1s are small and can easily be applied using capacity-limited viral vectors. In addition, normal regulation of gene expression is maintained in the natural chromosomal context. The endogenous promotor of the affected gene, pre-mRNA processing, and normal ratios of splice isoforms are maintained. Even in the case of a dominant-negative mutation, the engineered U1 approach would not only increase the functional gene product, but also reduce the detrimental mutated protein.

Engineering U1 to directly bind to the mutated splice donor site enabled us to correct splice defects in various target genes.^20^, ^21^, ^22^, ^23^, ^24^^,^^39^ In addition, we also generated a set of U1s binding to intronic sites in near proximity to, but not overlapping, the mutation. It can be speculated that the risk of side effects from the treatment, e.g., off-target missplicing, may be reduced by directing the therapeutic construct toward a less conserved intronic region. In this context, it has previously been proposed that the reason for the reduced risk for off-target effects may be due to higher target specificity.^25^ Nevertheless, it is important to consider that U1 binds with a maximum of only 9 bp to its target and, thus, shows only weak affinities and low specificity to its target site. To support the splicing mechanism, the U1-mediated effects require additional collaborating factors to recognize splice donor sites and to initiate the splicing process.^40^^,^^41^ Consequently, our results rather provide support for the hypothesis that a U1 binding in near proximity to splice donor sites is sufficient to successfully recruit the splicing machinery and to initiate the splicing process at the correct exon/intron boundary. Of note, the engineered U1 directly targeting the mutated splice donor site did not show any detectable therapeutic effects. Importantly, the MAXENT-based prediction was able to identify the engineered U1 binding site in the mutated *OPA1* transcript. This site did not act as a cryptic splice site, since neither mutation of the canonical splice site nor binding of engineered U1 resulted in transcripts employing this or alternative sites for splicing (Figure 7A). This suggested that bioinformatic predictions help to identify the therapeutic target sites for engineered U1s and that the best target site may occasionally be independent of the canonical splice donor site.

As a proof-of-concept study, our data showed for the first time the feasibility of splice-site correction as a treatment option for ADOA. To the best of our knowledge, our study is the first to demonstrate in primary human cell lines a superior treatment effect of an engineered U1 located at the intronic proximity of the mutation compared with an engineered U1 located at the mutated splice donor site (in contrast to what has recently been reported by, e.g., Balestra et al.^42^). These findings also suggest that splice donor sites may exist for which the U1 shows a preference to bind to near-intronic binding sites for exon definition. Alternatively, U1 binding in the proximity may suffice to recruit U6 to the splice donor site.^43^ Martinez-Pizarro and colleagues have shown by minigene assays that targeting U1 to intronic sites can aid in exon definition in phenylalanine hydroxylase (*PAH*) exon 11, which displays a weak 3′ splice site (MAXENT score 3.16) and is vulnerable to exonic mutations.^44^ Interestingly, *OPA1* exon 10 is not a known mutation hotspot in ADOA patients nor does it possess a weak 3′ splice site (MAXENT score of 7.19). In wild-type cells, *OPA1* is constitutively spliced, and residual exon skipping has not been observed by us nor described in the literature.

Increasing the amount of correctly spliced *OPA1* transcripts by viral vector-mediated transduction of U1 has the potential to overcome haploinsufficiency, which is the major disease pathomechanism in ADOA. Haploinsufficiency genes are generally characterized by a narrow expression range.^45^ According to the dosage-stabilizing hypothesis, small changes can lead to significant changes in fitness.^45^ In a model of Dravet syndrome, a 70% reduction in seizure frequency and severity was observed due to a 25% increase in Scn1a expression in the brain.^46^ Hence, we speculate that the U1-based treatment may lead to an amelioration of the ADOA phenotype and/or to a slower disease progression. Our findings further support that engineered splice factors offer a novel treatment option for blinding diseases. Further work is needed to address the question of safety and potential side effects. Obvious side effects were not observed in the primary patient-derived fibroblasts. Both treated and untreated cells showed comparable cellular morphology and growth behavior. Additional experiments are needed to check whether the risk of side effects is lower when engineered U1s are directed toward non-conserved sequences within introns. Furthermore, it remains to be established if correcting splice-site mutations is a feasible treatment approach in patients, and whether the increase in correctly spliced transcripts will be sufficient to overcome haploinsufficiency or to reduce disease severity and progression in ADOA patients. In this study, we used the lentivirus to transduce patient-derived cell lines. As a gene therapeutic approach in clinical settings, adeno-associated viruses (AAVs) expressing engineered U1 snRNAs, like U1_+18, might be preferred over lentiviruses. AAVs can be applied intravitreally to the patients' eyes to target the degenerating retinal ganglion cells, e.g., in Leber hereditary optic neuropathies.^47^^,^^48^

In conclusion, treatment of *OPA1* splice defects using engineered U1 offers a novel, promising therapy option for ADOA.

## Materials and methods

### Patients

This study was performed in accordance with the tenets of the Declaration of Helsinki. The collection of human skin biopsies and the use of human dermal fibroblasts were approved by the local ethics committees (Hannover Medical School, Germany [2576-2015], Faculty of Medicine and Health Sciences at the Carl-von-Ossietzky University Oldenburg, Germany [2018-097], and Ludwig-Maximilian University of Munich, Germany [45-14]). The nature and potential consequences of the study were explained to patients and unaffected controls, and written informed consent was obtained.

### Clinical characterization

The 34-year-old index patient was referred to the clinic with hereditary optic atrophy with no confirmed genetic diagnosis. Clinically, we also assessed her affected brother and her affected mother, and one unaffected first cousin, including neurological and general examination. An ophthalmological assessment was performed. Further details may be found in Table S1 and in the supplementary information.

### Genetic testing

Genotyping analysis by Sanger sequencing was performed in the index patient in order to confirm the *OPA1* genetic variant identified in the affected mother and the affected brother. The mutation was initially found by genetic diagnostic testing. Complete sequencing of the mitochondrial DNA was also performed for the index patient to exclude other relevant pathogenic sequence variants.

### Cell culture of patient-derived fibroblasts and HEK cells

After informed consent from each individual, a skin punch biopsy from the left upper arm was performed for the index patient (IV.2), her affected brother (IV.3), and her healthy cousin (IV.7).

Primary human skin fibroblasts from patients and controls were prepared from the skin biopsies as described previously,^21^ and cultivated in T75 flasks with minimal essential medium (MEM; Biowest, Renningen, Germany) supplemented with 20% fetal bovine serum (Biowest), 1.3% L-glutamine (Biowest), and 0.8% penicillin/streptomycin at 37°C in a humidified incubator with 5% CO_2_. HEK293T cells were cultured in DMEM (Biowest) supplemented with 10% fetal bovine serum (Biowest), 1% L-glutamine (Biowest), and 1% penicillin and streptomycin (Biowest) at 37°C in a humidified incubator with 5% CO_2_.

### Lentiviral transduction

The production of lentiviral shuttles was performed to transduce patient-derived cell lines and to express wild-type and engineered U1 (similar to that previously described in Glaus et al.^21^, ^22^). In brief, the human U1 cassette^49^ was cloned into the *HpaI* restriction site of the lentiviral plasmid p.RRLSIN.cPPT.SFFV/GFP.WPRE.^50^^,^^51^ U1 was fully adapted to the mutated splice donor site of exon 10 and to different positions within intron 10 of *OPA1* using the following primers for mutagenesis:

U1opaIn10−3_f GCCCAAGATCTCATATTTACATCGCAGGGGAGATACCATG

U1opaIn10−3_r CATGGTATCTCCCCTGCGATGTAAATATGAGATCTTGGGC

U1opaIn10+18_f GCCCAAGATCTCATATCAACCTTGCAGGGGAGATACCATG

U1opaIn10+18_r CATGGTATCTCCCCTGCAAGGTTGATATGAGATCTTGGGC

U1opaIn10+24_f GCCCAAGATCTCATACACATATCGCAGGGGAGATACCATG

U1opaIn10+24_r CATGGTATCTCCCCTGCGATATGTGTATGAGATCTTGGGC

U1opaIn10+28_f GCCCAAGATCTCATAATTACACAGCAGGGGAGATACCATG

U1opaIn10+28_r CATGGTATCTCCCCTGCTGTGTAATTATGAGATCTTGGGC

U1opaIn10+47_f GCCCAAGATCTCATAAGCTCATCGCAGGGGAGATACCATG

U1opaIn10+47_r CATGGTATCTCCCCTGCGATGAGCTTATGAGATCTTGGGC

For production of lentiviral particles, HEK293T cells were seeded at 6–7 × 10^6^ cells per T75 flask. On the next day, co-transfection was performed with the packaging plasmids pSPAX2 (13 μg) and pMD2.G (4 μg) and the expression plasmid containing the adapted U1 cassette (24 μg) using 75 μg branched polyethyleneimine (Sigma-Aldrich, Munich, Germany) as the transfection reagent. After 6 h incubation at 37°C with 5% CO_2_, the transfection medium was replaced by fibroblast medium for virus production. Virus-containing medium was harvested after 1 and 2 days, pooled, and stored at 4°C. For lentiviral transduction, fibroblasts were seeded in T25 flasks at 300,000 cells per flask in virus containing MEM and cultured for 72 h. Fibroblasts were regularly checked for eGFP expression to monitor successful transduction.

### RNA isolation and RT-PCR analysis

Total RNA from cell pellets was isolated using the NucleoSpin RNA isolation kit (Macherey-Nagel, Düren, Germany) according to the manufacturer's instructions. First-strand cDNA synthesis was performed using random primers and SuperScript III (Thermo Fisher, Germany). For RT-PCR, HotFire Taq polymerase (Solis BioDyne, Tartu, Estonia) was used with primers hOPA1_for, 5′-GATGACAAAGGCATTCATCA-3′, and hOPA1_rev, 5′-GTTTCCTTTGTGTCAGGAGC-3′, located in exons 7 and 13 of *OPA1*, respectively. PCR products were resolved on a 2% agarose gel and visualized using ROTI GelStain (Carl Roth, Karlsruhe, Germany). Relative quantification of band intensities was performed with Image Lab software (Bio-Rad, Feldkirchen, Germany).

### Western blot

Cells were harvested and washed with PBS, and the cell pellet was resuspended in lysis buffer (10 mM Tris-HCl [pH 7.5], 150 mM NaCl, 4% glycerol, 1% Triton X-100, 0.1% sodium deoxycholate, 0.05% SDS) supplemented with protease inhibitors (S8830, Sigma-Aldrich). After 30 min incubation on ice, the supernatant was cleared from insoluble debris by centrifugation (30 min, 15,000*g*, 4°C). Forty micrograms of total protein lysate was loaded per lane, separated by 12% SDS-PAGE, and blotted onto a polyvinylidene fluoride (PVDF) membrane. After being blocked with 5% BSA/TBST for 1 h at room temperature, the membrane was incubated overnight at 4°C with an OPA1-specific antibody (1:1,000 diluted, mouse anti-OPA1 clone 18, cat. no. 612,607; BD Biosciences, Heidelberg, Germany) in blocking buffer. As loading control, a mouse anti-GAPDH antibody (Merck, Chemicon, MAB374) was used. A peroxidase-conjugated goat anti-mouse antibody (NB7539, Novus) was used as secondary antibody, followed by enhanced chemiluminescence (ECL) detection. Relative protein levels were calculated based on band intensity quantifications using ImageLab software (Bio-Rad).

### Structural modeling

Based on the crystal structure of the OPA1 GTPase domain (PDB: 6JTG^31^) structural changes caused by exon 10 skipping in *OPA1* were modeled by homology modeling using the Swiss-Model server (https://swissmodel.expasy.org/)^52^ at the Swiss Institute of Bioinformatics.

### Statistical analysis

Experiments were replicated at least three times with independently cultured and treated cells unless otherwise specified. Data are presented as the mean ± standard deviation (SD). Error bars indicate the SD. Statistical significance was calculated using Welch's two-sample t test with non-pooled SD (∗∗∗p < 0.001; ∗∗p < 0.01; ∗p < 0.05; n.s., not significant, p > 0.05). All statistical tests were two-sided and performed using the R software package (version 3.6).

## Supplemental information

Document S1. Supplemental text, Figures S1–S4, and Table S1

Document S2. Article plus supplemental information

## Footnotes

Supplemental information can be found online at https://doi.org/10.1016/j.omtn.2021.10.019.
