# Supplementary material for: Autosomal dominant optic atrophy: A novel treatment for OPA1 splice defects using U1 snRNA adaption
Source: Mol Ther Nucleic Acids. 2021 Oct 21;26:1186–97. doi: 10.1016/j.omtn.2021.10.019 (PMC8604756; doi:10.1016/j.omtn.2021.10.019)
Supplement: Document S1. Supplemental text, Figures S1–S4, and Table S1 [file mmc1.pdf]

OMTN, Volume *26*

Supplemental information

Autosomal dominant optic atrophy: A novel treatment for *OPA1* splice defects using U1 snRNA adaption

Christoph Jüschke, Thomas Klopstock, Claudia B. Catarino, Marta Owczarek-Lipska, Bernd Wissinger, and John Neidhardt

Supplemental Information

Supplemental text

Clinical characterization of all affected family members

Index Patient IV.2

First seen in clinic at 34 years of age. Eldest of two siblings in a non-consanguineous family from Germany. Clinical onset in childhood in primary school and the evolution with slowly progressive bilateral visual loss, with a visual acuity at 34 years of age of 0.25 bilaterally. No other relevant medical history. No concomitant medication. Non-smoker. General examination was unremarkable. Neurological examination showed bilateral visual loss (BCVA: 0.25 bil.), temporal predominant optic pallor and exophoria in cover-test.

Patient IV.3

Younger brother of the index patient, first seen in clinic at 32 years of age. Clinical onset was in early childhood, in in preschool age (kindergarten), “always had eye problems”. At the age of 5 years an operation was performed because of strabismus. The evolution was a slowly progressive bilateral visual loss, with a visual acuity at 32 years of age of OD 0.20 and OS 0.16. Also, the patient complains of colour vision problems. Colour vision defects and centrocecal visual field defect with reduced thickness of the retinal nerve fibre layer in the OCT of the papilla were documented. The patient uses zooming and gets closer at about 10 cm to be able to read. Relevant medical history includes a surgery for strabismus in 1978. In 2006, the patient had a surgery with the removal of a pituitary adenoma. He is non-smoker. General examination was unremarkable. Neurological examination showed bilateral visual loss, temporal predominant optic pallor and exophoria.

Patient III.2

Mother of the index patient, first seen in clinic at 76 years of age. Clinical onset at age 15 years, with a slowly progressive bilateral visual impairment over the years. An optic atrophy was documented. A wrong diagnosis of Leber's hereditary optic neuropathy was proposed but never genetically confirmed. The diagnosis was changed to OPA1-ADOA in June 2012. Genetic diagnostic testing using a gene panel for Optic atrophy detected the heterozygote *OPA1* mutation c.1065+5G>A, p.? in intron 10, classified initially as variant of unclear significance. The patient started taking Idebenone 150 mg 1x/d, in the Summer of 2015, and was seen in clinic six months after starting the treatment. Additionally, she showed spastic paraparesis and sensorimotor polyneuropathy with sensory ataxia. Relevant medical history also includes a monoclonal gammopathy-IgG-lambda, lumbar spinal stenosis with L4/5 spondylolisthesis und kyphoscoliosis, migraine with aura and osteoporosis. Neurological examination showed bilateral vision loss, spastic paraparesis, hyperreflexia and extensor plantar response bilaterally, reduced bimalleolar vibration sense, and spastic-ataxic gait.

Patient V.2

Patient V.2 was the younger son of the index patient, which was not seen in clinic. Clinical onset was reported to have occurred at an age of 1.5 years. He presented with progressive bilateral visual loss. He reads and plays piano, but needs to be very close to the written text. Best corrected visual acuity was OD 0.50 and OS 0.40 at an age of 6 years. With 7 years of age 0.30 was detected bilaterally, with a temporal predominant optic pallor and exophoria in cover test.

Patient III.3, deceased

The brother of the mother of the index patient was not seen in clinic. An optic atrophy was diagnosed. Later, he was affected by parkinsonism.

Patient III.4, deceased

The brother of the mother of the index patient was not seen in clinic, but was diagnosed with optic atrophy. A stroke and a myocardial infarct occurred at an age of 83 years.

Patient II.2, deceased

II.2 is the maternal grandmother of the index patient and was not seen in clinic. She was reported to have had visual impairment, as well as parkinsonism and dementia.

Table S1. Clinical characteristics of the family.

Subject ID	Gender	Year of birth	Age at study (y)	Age at onset of vision loss (y)	ADOA symptoms Y/N	Previously recorded BCVA (OD)	Previously recorded BCVA (OS)	Last recorded BCVA (OD)	Last recorded BCVA (OS)	Comorbidities	Genetic testing	Skin biopsy
IV.2 (Index)	F	1972	34	Primary school age	Yes	0.25 @32y	0.25 @32y	0.25 @34y	0.25 @34y	None	Mutation search by Sanger; mtDNA-Seq	Yes
IV.3	M	1974	32	Preschool age	Yes	0.20 @15y	0.30 @15y	0.20 @30y	0.16 @30y	Pituitary adenoma resected	Mutation search by Sanger	Yes
V.2	M	2009	NA	1.5	Yes	0.50 @6y	0.30 @6y	0.30 @7y	0.30 @7y	None	NA	No
IV.7	M	NA	NA	NA	No	Normal	Normal	Normal	Normal	None	Mutation search by Sanger	Yes
III.2	F	1940	76	15	Yes	NA	NA	NA	NA	Spastic paraparesis, sensorimotor polyneuropathy; monoclonal gammopathy-IgG-lambda, lumbar spinal stenosis, migraine	LHON mutation search; gene panel OA	No
I.1	M	NA	NA	NA	NA	NA	NA	NA	NA	NA	NA	No
II.2	F	NA	NA	NA	NA	NA	NA	NA	NA	Parkinsonism and dementia	NA	No
III.3	M	NA	NA	NA	NA	NA	NA	NA	NA	Parkinsonism	NA	No
III.4	M	NA	NA	NA	Yes	NA	NA	NA	NA	Stroke and myocardial infarct at 83 y	NA	No

Abbreviations: BCVA, best corrected visual acuity; NA, not applicable or not available; OA, optic atrophy; OD, right eye; OS, left eye; y, years.

Supplemental figures

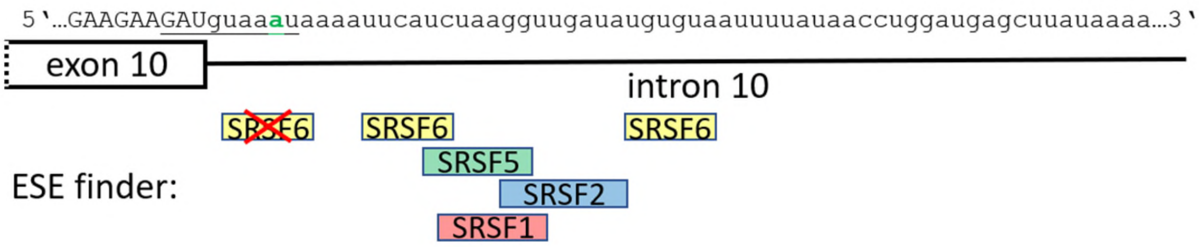

Figure S1. *In-silico* prediction of SRSF protein binding sites. Schematic representation of the region around the splice donor site of *OPA1* exon 10 / intron 10. Potential binding sites for SRSF proteins are predicted by ESE finder (http://rulai.cshl.edu/cgi-bin/tools/ESE3/esefinder.cgi?Process=home; [1]) and indicated by colored boxes. The c.1065+5G>A mutation abolishes one SRSF6 site (red cross). Nucleotides of the splice donor site are underlined; the *OPA1*: c.1065+5G>A mutation is highlighted in green.

```
OPA1_Danio            SDYDSNYNTQDHLPRVVVVGDQSAGKTSVLEMIAQARIFPRGSGEMMTRSPVKVTLSEGP
OPA1_Gallus           SDYDASYNTQDHLPRVVVVGDQSAGKTSVLEMIAQARIFPRGSGEMMTRSPVKVTLSEGP
OPA1_Rattus           SDYDASYNTQDHLPRVVVVGDQSAGKTSVLEMIAQARIFPRGSGEMMTRSPVKVTLSEGP
OPA1_Pan              SDYDASYNTQDHLPRVVVVGDQSAGKTSVLEMIAQARIFPRGSGEMMTRSPVKVTLSEGP
OPA1_Homo             SDYDASYNTQDHLPRVVVVGDQSAGKTSVLEMIAQARIFPRGSGEMMTRSPVKVTLSEGP
OPA1_Homo_patient     SDYDASYNTQDHLPRVVVVGDQSAGKTSVLEMIAQARIFPRGSGEMMTRSPVK-------
                      ****:.***********************************************

OPA1_Danio            HHVAMFKDSSREFDLGKEEDLAALRHEIELRMRKSVKEGQTVSPETISLSVKGPGIQRMV
OPA1_Gallus           HHVALFKDSSREFDLTKEEDLAALRNEIEIRMRNSVKEGCTVSTETISLSVRGPGLQRMV
OPA1_Rattus           HHVALFKDSSREFDLTKEEDLAALRHEIELRMRKNVKEGCTVSPETISLNVKGPGLQRMV
OPA1_Pan              HHVALFKDSSREFDLTKEEDLAALRHEIELRMRKNVKEGCTVSPETISLNVKGPGLQRMV
OPA1_Homo             HHVALFKDSSREFDLTKEEDLAALRHEIELRMRKNVKEGCTVSPETISLNVKGPGLQRMV
OPA1_Homo_patient     --------------------LAALRHEIELRMRKNVKEGCTVSPETISLNVKGPGLQRMV
                                          *****:***:***:.**** *** *****.*:***:****

OPA1_Danio            LVDLPGVISTVTTGMAADTKETIFSISKAYMQNPNAIILCIQDGSVDAERSIVTDLVSQM
OPA1_Gallus           LVDLPGVISTVTSGMAPDTKETIFSISKAYMQNPNAIILCIQDGSVDAERSIVTDLVSQM
OPA1_Rattus           LVDLPGVINTVTSGMAPDTKETIFSISKAYMQNPNAIILCIQDGSVDAERSIVTDLVSQM
OPA1_Pan              LVDLPGVINTVTSGMAPDTKETIFSISKAYMQNPNAIILCIQDGSVDAERSIVTDLVSQM
OPA1_Homo             LVDLPGVINTVTSGMAPDTKETIFSISKAYMQNPNAIILCIQDGSVDAERSIVTDLVSQM
OPA1_Homo_patient     LVDLPGVINTVTSGMAPDTKETIFSISKAYMQNPNAIILCIQDGSVDAERSIVTDLVSQM
                      ********.***:*** *******************************************
```

Figure S2. Multiple species alignment of OPA1 protein sequences. Alignment of OPA1 from different species (using Clustal Omega). OPA1 exon 10 is part of the highly conserved OPA1 GTPase domain. The region of exon 10 is indicated by a blue bar. Danio = zebrafish (NP_001007299.1), Gallus = chicken (NP_001034398.1), Rattus = rat (NP_598269.3), Pan = chimpanzee (XP_024211635.1), Homo = human (NP_056375.2). * (asterisk) = single, fully conserved residue; : (colon) = conservation between groups of strongly similar properties; . (period) = conservation between groups of weakly similar properties.

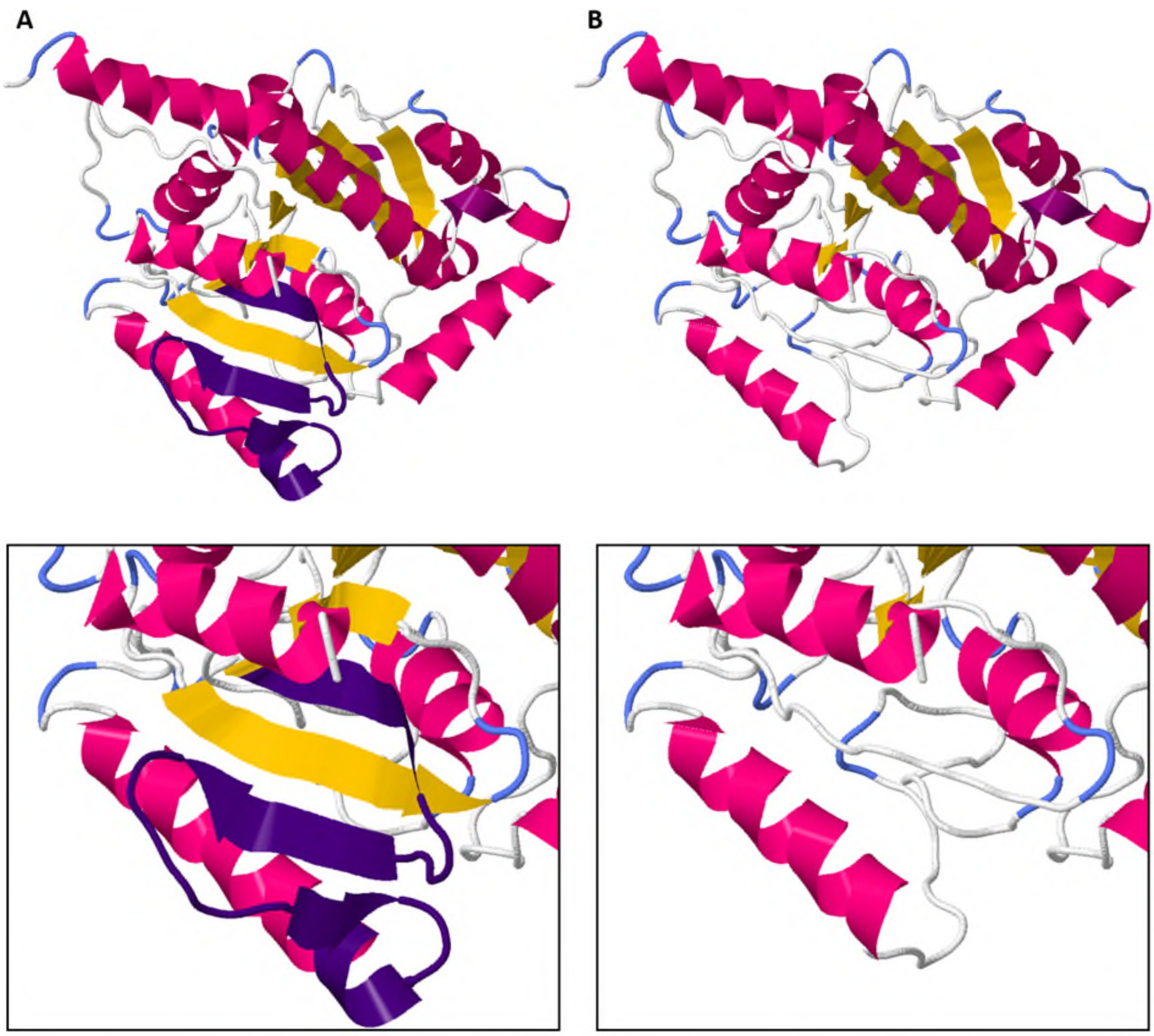

Figure S3. Homology models of the GTPase domain comparing the reference OPA1 with the mutated OPA1 lacking exon 10. (A) Structural presentation of the OPA1 minimal GTPase domain (PDB 6JTG; [2]). Amino acids encoded by exon 10 are shown in indigo. (B) Homology model representation (Swiss-Model server) predicting the OPA1 minimal GTPase domain lacking 27 amino acids encoded by *OPA1* exon 10. Alpha helices are shown in red, beta strands are shown in yellow.

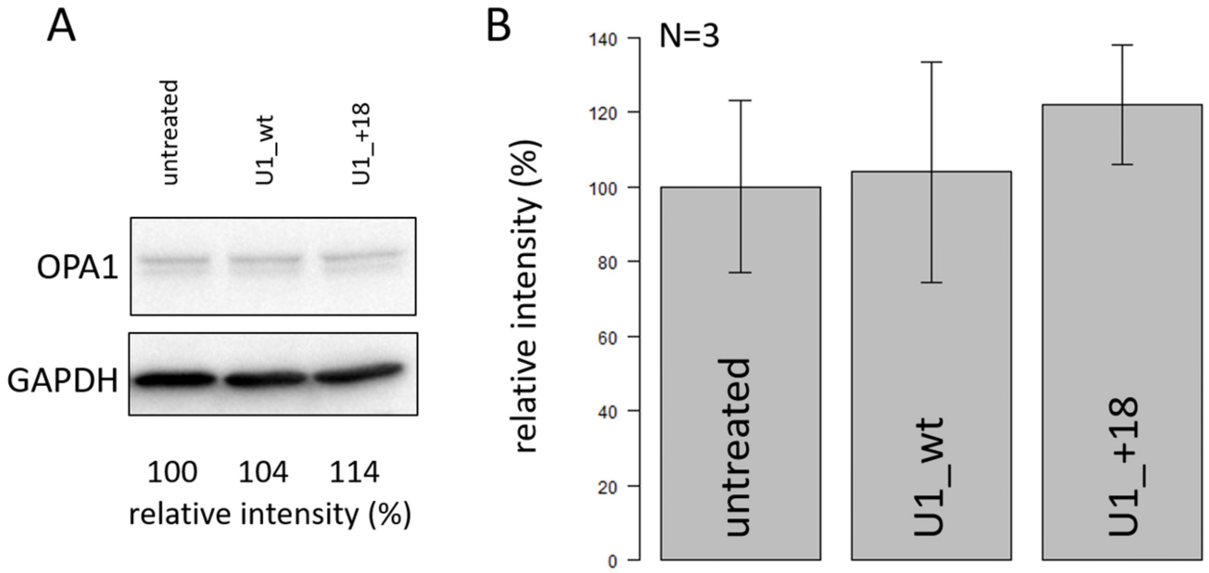

Figure S4. Quantification of OPA1 protein expression after treatment with engineered U1 snRNAs. (A) Representative western blot of OPA1 levels in primary fibroblasts from patient VI.2 after treatment with lentiviral shuttles expressing the engineered U1_+18 or U1_wt. OPA1 appears as two bands, L-OPA1 and S-OPA1. GAPDH was used as a loading control. (B) Relative quantification of OPA1 protein levels by densitometric analyses. A tendency towards increased OPA1 protein expression was associated with the treatment applying engineered U1 snRNA U1_+18. The levels of OPA1 were normalized against GAPDH. Error bars indicate the SD from three biological replicates (N=3). The differences between samples are not statistically significant, $P > 0.05$ (pairwise t-tests with non-pooled SD).

References

1. Cartegni, L, Wang, J, Zhu, Z, Zhang, MQ, and Krainer, AR (2003). ESEfinder: A web resource to identify exonic splicing enhancers. *Nucleic Acids Res* **31**: 3568-3571.
2. Yu, C, Zhao, J, Yan, L, Qi, Y, Guo, X, Lou, Z, Hu, J, and Rao, Z (2020). Structural insights into G domain dimerization and pathogenic mutation of OPA1. *J Cell Biol* **219**.
